# A periodic-like table of space groups

**DOI:** 10.1107/S2056989023000786

**Published:** 2023-02-07

**Authors:** Bart Kahr

**Affiliations:** a Department of Chemistry and Molecular Design Institute, New York University, 29 Washington Place, New York City, NY 10003, USA; Universidad de Los Andes Mérida, Venezuela

**Keywords:** periodic table, space groups, point groups, symmorphic, Fedorov, Schoenflies, Barlow

## Abstract

There are about 100 chemical elements, and 200 space groups, rounding to the nearest hundreds. The elements, by virtue of the iconic periodic table, which hangs in schoolrooms worldwide, are part of our common culture. Few on the outside of solid-state science and discrete mathematics know of the remarkable enumeration of the space groups and how they organize solids, the chemical elements included, that can be conformed to translationally periodic lattices in three dimensions. Perhaps the comparative inaccessibility of space groups to non-specialists can be attributed to the absence of a single chart that captures and organizes the space groups in a glance, as does the periodic table for the chemical elements? Here is designed a periodic-like table of the space groups. The symmorphic space groups are organized as the coordinates of two non-linear, non-orthogonal axes that capture the point group symmetry and the order of the group, the multiplicity of general positions. The related non-symmorphic groups are treated as ‘isotopes’ of the symmorphic groups and are enumerated with a subscript. The intent is to inspire better designs and to bring the crystallographic space groups to the attention of a wider audience.

## Abstract 2

A periodic-like table of the 230 space groups, a sweeping overview in the spirit of the periodic table of the chemical elements, is presented. It organizes the 73 symmorphic space groups along two non-orthogonal axes of point group symmetry and general position multiplicity that separates the crystal systems in discrete color fields. The remaining non-symmorphic groups, for reasons of graphical economy, are enumerated as ‘isotopes’ of their parent symmorphic groups.

## 1. Introduction

Dmitri Mendeleev (1834–1907), the youngest of a great number of children (as few as 11 or as many as 17 depending on the source), received a consequential education only because his mother rode with him from Siberia to Moscow and then to St Petersburg, a journey of 2900 kilometres. Dmitri and Maria Mendeleeva stopped first at the University of Moscow, but Dmitri was not admitted. They kept going to the University of Saint Petersburg and again Dmitri was refused a place. He ultimately enrolled in the lesser Main Pedagogical Institute, separate from Saint Petersburg University but housed within its grounds (Gorin, 2004 ▸).

Without the determination of Maria Mendeleeva and the formal education of her youngest child (Larcher, 2019 ▸), the periodic table that organizes the elements, an iconic chart hanging in classrooms the world over, might not have come to us as soon as it did and in quite the same way. However, Mendeleev’s big journey and his big chart are just part of the drama. The discovery of the elements, some 118 and counting, begins in prehistory and carries us through the atomic age. It is a story rich in ingenuity, persistence, and courage told in successive generations, as it gathers new chapters from scientists who are synthesizing super heavy elements (Chapman, 2019 ▸).

Books about the elements and the periodic table are legion. Gray’s artful *The Elements* (Gray, 2012 ▸) has sprouted periodic table posters, placemats, puzzles, and iPad apps, satisfying a considerable appetite. The number of volumes devoted to the discovery of the elements (Ball, 2021 ▸) or their properties (Elmsley, 2001 ▸) or the periodic table (Scerri, 2007 ▸), just in print in English, are too great to list. There are offerings appropriate to grade schoolers (Zovinka & Clark, 2020 ▸) middle schoolers (McHenry, 2021 ▸), as well as adults who were inattentive middle-school chemists but remember the awkward/looming/inscrutable chart (Røyne, 2020 ▸). It is a story that has been told by our best writers, and it forms the skeletons of a pair of essential autobiographies (Levi, 2002 ▸; Sacks, 1988 ▸).

There is another scheme for organizing solid matter that involved the invention and organization of a set of 230 things, the classical symmetry groups of crystals. Three scientists working independently in Russia, Germany, and England, discovered the symmetries or so-called space groups that underlie the architecture of solids. Fedorov (1853–1919) (Fedorov, 1890 ▸, 1971 ▸), Schoenflies (1853–1928) (Schoenflies, 1891 ▸; Hinton, 1963 ▸), and to a lesser extent Barlow (1845–1934) (Barlow, 1894 ▸; Mauskopf, 2015 ▸) arrived at this set independently after 1890 – we are setting aside quasiperiodic crystals, not invented and/or discovered until the 1980s (Steinhardt, 2019 ▸) – but their motivations, experiences, and solutions were remarkably different.

Fedorov was a member of the staff of the Geological Commission. Scouring the Ural Mountains for mineral resources was his principal occupation. Fedorov was long interested in symmetry after devouring his elder brother’s military school elementary geometry primer. He became obsessed with the regularity of nature and the unity of science. Before solving the space-group problem, Fedorov sought to explain the periodicity of the chemical elements with a planetary model of elementary atomic particles. In fact, he shared these ideas with Mendeleev himself (Galiulin, 2003 ▸). To geometry was added the crystals beneath his feet. From this combination the space groups emerged.

Schoenflies was a mathematics professor in Göttingen (Kaemmel, 2006 ▸). He completed his habilitation in 1884, just before Felix Klein (1849–1925) arrived to begin his transformation of Göttingen into a premier center for mathematics. Schoenflies was mentored by Klein. For him, the discovery of the space groups was an exercise in geometrical group theory.

Barlow was a gentleman scientist, having inherited a fortune from his father, a London real estate developer. He pursued crystal structure as a hobby. His formal training was limited, paradoxically an advantage according to his collaborator Pope because it left him ‘unhindered by authority’ (Pope, 1935 ▸). Barlow’s solution is therefore the most idiosyncratic. He arranged gloves in space suspended on posts within frames (Tandy, 2004 ▸; Paufler, 2019 ▸).

Finding the 230 space groups was an abstract exercise. At the time (1890s), there was no way to locate positions of atoms and molecules in solids. No crystal structures had been determined. X-rays had yet to be discovered.

**Figure 1:**
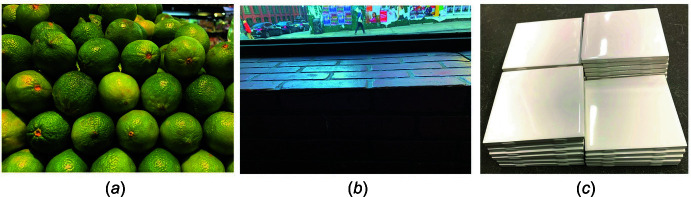
Three macro-world illustrations that were found during one afternoon in Brooklyn. (*a*) *Fm* 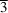 *m* limes, (*b*) *Cmmm* bricks, (*c*) *P*4*mm* ceramic tiles. Gravity has a way of squeezing out groups that are in the main organized on glide-planes or screw axes.

Some space groups can describe a small number of common objects encountered in the wider world, and so are easier to conceptualize. Fruit sellers routinely illustrate the two space groups that describe the closest packing of spheres, *Fm* 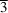 *m* [Fig. 1 ▸(*a*) ▸] and *P*6_3_/*mmm*. And these two space groups are the most well populated among crystals of the chemical elements (Donohue, 1974 ▸). Isolated atoms are spherical, and many behave as hard spheres when packed. Children’s building blocks, if cubes, can be assembled into self-supporting *Pm* 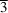 *m* structures or *Pmmm* if rectangular (simple orthorhombic). *Cmmm* [base-centered orthorhombic, Fig. 1 ▸(*b*) ▸] arises as in brickwork or *P*2/*m* for rows successively displaced one above the other. The storeroom of a tile shop might be a good place to find an approximation of simple tetragonal *P*4*mm* [Fig. 1 ▸(*c*) ▸] or hexagonal *P*6*mm* space groups. But, where in everyday life do we encounter the diamond lattice, *Fd* 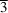 *m*, or *P*2_1_2_1_2_1_? By comparison, Mendeleev predicted places for four undiscovered elements, but 60 others were already known. The space groups were enumerated *de novo* without the surety of even one crystal structure.

Fedorov did not expect to live to see the confirmation of his greatest work, but he did. In 1890, William Lawrence Bragg (d. 1971) was born in Adelaide, Australia (Jenkin, 2006 ▸). He would invent the technique with his father that would ultimately substantiate the predictions of Fedorov, Schoenflies, and Barlow (Authier, 2013 ▸).

Enumeration of the space groups is a remarkable example of systematic geometric thinking (Burckhardt, 1967 ▸). Nevertheless, the space groups are hardly appreciated outside the world of solid-state science where their symmetries organize the daily work of physicists, chemists, geologists, metallurgists, and structural biologists (Burns & Glazer, 1990 ▸). The uninitiated may be surprised that amongst the seeming infinite diversity of organized structures in our world, there are a finite number of qualitatively different ways of arranging identical copies of an asymmetric object (*e.g.*, a potato) in three-dimensional space. As the elements assemble with themselves or one another, they are constrained by these 230 arrangements (again excepting quasi-periodic crystals).

Can this lack of familiarity be remedied? Is there some value to such a remediation? Perhaps, a sweeping view of the space groups in the manner of the periodic table of the chemical elements is missing? Perhaps we need a *periodic-like table of space groups*?

## 2. A periodic-like table of space groups

According to Senechal (1990 ▸), ‘The presentation of the space groups in a form useful to X-ray crystallographers became an urgent problem as soon as the importance of the space groups for structure determination was recognized.’ She identifies Niggli (Niggli, 1919 ▸), Wyckoff, (Wyckoff, 1922 ▸) and Astbury and Yardley (Astbury & Yardley, 1924 ▸) as those who first set to work. They produced lists that ultimately matured into the *International Tables for Crystallography, Volume A* (Hahn, 2006 ▸).

The job of creating a periodic-like *table* of space groups is invitingly well bounded. The periodic table of the elements has seven rows; there are seven crystal systems. The first row of the periodic table has two elements, H and He; the first crystal system has two space groups, *P*1 and *P* 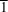 . There are 32 crystallographic point groups and 32 columns in the periodic table when considering the lanthanides and actinides in their proper places (2*s* + 6*p* + 10*d* + 14*f* = 32 electrons). Here, there is a good starting point with the least symmetric groups, and one of the dimensions of the space-group table. The 32 point groups, first identified as a closed set by Frankenheim and independently by Hessel, must map cleanly onto the seven crystallographic systems later identified by Bravais (Burke, 1966 ▸). Now, it is a matter of choosing the other dimension.

**Figure 2:**
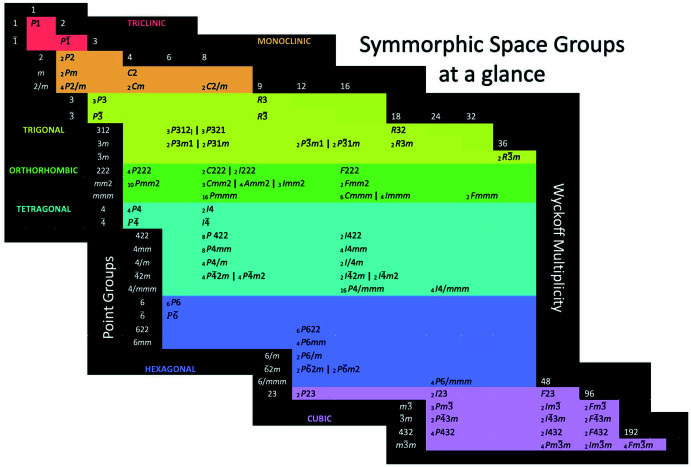
If the *International Table for Crystallography, Volume A* (Hahn, 2006 ▸) were reduced to one page it might resemble a periodic-like table of space groups. Point-group symmetry and Wyckoff multiplicity are plotted along non-orthogonal axes, separating the crystal systems into colored fields.

If we choose the second dimension as the number of general positions of the space group, an essential attribute for any practical crystal analysis, we can plot two non-orthogonal dimensions that progress from the least symmetric groups (1 general position in *P*1) to the most symmetric (192 in *Fm* 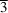 *m*.) The 230 space groups fall into 16 sets depending on the number of general positions: 1, 2, 3, 4, 6, 8, 9, 12, 16, 18, 24, 32, 36, 48, 96, and 192. In choosing any coordinate in the table, say *I*422, roughly in the middle, we read left to get the point group (422), and up to get the order (16). Fig. 2 ▸ plots the 32-element coordinate as rows as opposed to columns in the periodic table of elements, but rows and columns could be easily interchanged. Such a design creates at a glance a table of the space groups that progresses from the upper left to the lower right and divides the crystallographic systems in seven continuous fields of color.

We emphasize our choice of the term ‘*periodic-like* table of space groups’ rather than ‘table of space groups’. The latter is too generic and does not capture or conjure the table of the elements, but we qualify ‘periodic’ because there is not a natural columnar progression or repeating characteristic.

Space groups are naturally related to one another as sub- and supergroups. *P*1 is subgroup (in the mathematical sense) of all other space groups. The hydrogen atom is a subgroup (in the colloquial sense) of all other atoms, However, while filling of spherical harmonic functions (orbitals) by electrons keeps the elements in their columns, trees of groups and supergroups are so entangled as to appear as an impossibly complex organizing principle for a two-dimensional organizational scheme (Müller, 2013 ▸). Sub- and supergroup relations cross rows and columns with abandon (Bärninghausen, 1980 ▸; Koch, 1984 ▸). See, for example, the output of the program *SUBGROUPGRAPH* (Ivantchev *et al.*, 2000 ▸) implemented on the Bilbao Crystallographic Server (https://www.cryst.ehu.es). The classification in Fig. 2 ▸ is something much simpler.

Only the 73 symmorphic space groups are plotted in Fig. 2 ▸. A symmorphic group may be specified by operations not involving translations all acting on a common point. Most symmorphic space groups have a number of associated non-symmorphic groups where operations of point groups in the full Hermann–Mauguin space-group symbol are replaced by combined rotation–translation operations, and reflection–translation operations. Non-symmorphic groups must be specified by at least one non-primitive translation. *Pm* and *Pc* represent a symmorphic and non-symmorphic pair with the same point symmetries (*m*) and an equal number of general positions (2). Some space groups are singular (*e.g. P*1, *R*3), whereas some symmorphic groups have as many as 15 non-symmorphic partners (*e.g. Pmmm*: *Pnnn, Pccm, Pban, Pmma, Pnna, Pmna, Pcca, Pbam, Pccn, Pbcm, Pnnm, Pmmn, Pbcn, Pbca, Pnma*). To account for these relationships, we have included a left subscript giving the total number of symmorphic groups and non-symmorphic groups with the same point symmetry. The absence of a subscript implies the absence of a non-symmorphic group and a ‘1’ is assumed. Subscripts sum to the total number of space groups, 230. The non-symmorphic groups function like isotopes in the periodic table; they are like one another, but not exactly so. Whereas the properties of most crystals are given by point symmetry, there are real-world manifestations of the differences between symmorphic and non-symmorphic groups, diffraction conditions notwithstanding. For instance, *P*2_1_ might grow in sub-steps of height *b*/2, rather than whole steps. That is, local properties might diverge from the Neumann–Curie Principle (Jaeger, 1917 ▸) but not those properties of crystals if taken as a whole. Non-symmorphic manifestations on crystal properties are typically small effects, like isotope effects.

## 3. Who would use this and how?

Keeping track of the space groups is a challenge for both experienced crystallographers with dogeared copies of the *International Tables, Volume A*, and newcomers to the field (Hahn, 2006 ▸). For older readers, recall when you had to keep track of the space groups for the first time decades ago, or for younger readers, keeping track of something that is not even bounded by boards of a book but is only familiar online (Fuess *et al.*, 2007 ▸). Here, we propose a chart that may help both experts and novices to feel grounded by capturing the ensemble of space groups in one sweep.

How might Fig. 2 ▸ be used in practice? Given a trigonal, piezoelectric crystal indexed with hexagonal axes, it is quickly apparent that the choices are either *P*3 or *P*3*m*1, *P*31*m*, *P*3*c*1 or *P*31*c*. The latter two can be eliminated on the basis of extinction conditions leaving enantiomorphous *P*3 and non-enantiomorphous *P*3*m*1 and *P*31*m*, the latter pair being a particularly subtle distinction. Or, given a chiral cubic crystal, a glance narrows our choices to *P*23, *P*2_1_3, *P*432, *P*4_1_32, *P*4_2_32, or *P*4_3_32. The distinction comes down to the possibility of a fourfold axis and the extinction conditions. Distinguishing among the enantiomorphs (*P*4_1_32 and *P*4_3_32) then requires a reliance on anomalous dispersion.

There are lots of potential periodic-like tables of space groups, just as there are many designs for the periodic table of elements (Mazurs, 1974 ▸). What is offered here for space groups is just one idea. We hope that it prompts the invention of better schemes. There are more comprehensive guides to space group hierarchies (Nespolo *et al.*, 2018 ▸) but they are not intended to function holistically as is Fig. 2 ▸. Does Fig. 2 ▸ have value? Is there something related that would be better still? We invite better designers to replace Fig. 2 ▸ with something enduring.

*Note added in proof.* There is an artful chart of the space groups that can be hung in a classroom. It is is an illustrated list of structures in order 1–230 (see Mayer, B., Johnson, L., Wyllie, D., Rodrigues, L. & Vasiliev, V. https://crystalsymmetry.wordpress.com/230-2/.
